# Efficacy of Trihexyphenidyl on Apraxia of Eyelid Opening in Parkinsonism: A Case Report

**DOI:** 10.7759/cureus.56232

**Published:** 2024-03-15

**Authors:** Koji Hayashi, Rei Asano, Mamiko Sato, Yuka Nakaya, Asuka Suzuki, Naoko Takaku, Kouji Hayashi, Yasutaka Kobayashi

**Affiliations:** 1 Department of Rehabilitation Medicine, Fukui General Hospital, Fukui, JPN; 2 Graduate School of Health Science, Fukui Health Science University, Fukui, JPN

**Keywords:** blepharospasm, focal-dystonia, trihexyphenidyl, parkinson disease treatment, apraxia of lid opening

## Abstract

Apraxia of eyelid opening (AEO) is occasionally seen in Parkinson’s disease (PD) or related diseases. However, many clinicians have trouble with the management of AEO by Parkinsonism. In this report, we describe a case of AEO in Parkinsonism improved by trihexyphenidyl (THP). The patient was a 64-year-old woman, who was previously healthy but developed bradykinesia. She was clinically diagnosed as PD due to an L-dopa challenge test, but no other detailed tests were performed. She started antiparkinsonian medications and her symptoms were improved at an early phase. However, her motor symptoms were gradually exacerbated over time, and antiparkinsonian medications were dosed up. At 69 years old, blepharospasm and AEO developed. Although other antiparkinsonian medications did not improve her AEO, THP cured AEO dramatically at 73 years old. In this report, we discuss a mechanism of AEO by Parkinsonism and the pathway of THP for the improvement of AEO.

## Introduction

Apraxia of eyelid opening (AEO) is defined as a non-motor abnormality characterized by the patient's difficulty elevating their eyelids bilaterally [1]. AEO is most commonly related to blepharospasm or focal dystonia of the eyelids [1]. In particular, it is related to idiopathic (including benign essential blepharospasm), infectious (including keratitis, blepharitis, dacryocystitis, conjunctivitis), toxic exposure (including extensive sunlight), autoimmune (including keratoconjunctivitis sicca from Sjogren's disease), and neurodegenerative (including Parkinson's disease (PD) and Huntington's disease) [1]. Among these underlying diseases, AEO due to PD or other Parkinsonism diseases, including progressive supranuclear palsy (PSP) and multiple system atrophy (MSA), is occasionally seen in terms of their prevalence and frequency of consultation. However, many clinicians have trouble with the management of AEO.

Trihexyphenidyl (THP) is an oral, centrally acting anticholinergic drug used mainly in the treatment of PD and movement disorders [2]. THP acts as an antagonist of central cholinergic receptors and helps balance cholinergic transmission in the basal ganglia [2]. In addition, THP may also block dopamine reuptake and storage in the central nervous system, leading to increasing dopaminergic activity [2]. Regarding other diseases, THP is used for the treatment of spastic disorders, extrapyramidal disorders due to medications, and dystonia [2,3]. Herein, we report a case of AEO caused by Parkinsonism improved by THP. In addition, we discuss a mechanism of AEO by Parkinsonism and the pathway of THP for the improvement of AEO in this report.

## Case presentation

**Figure 1:**
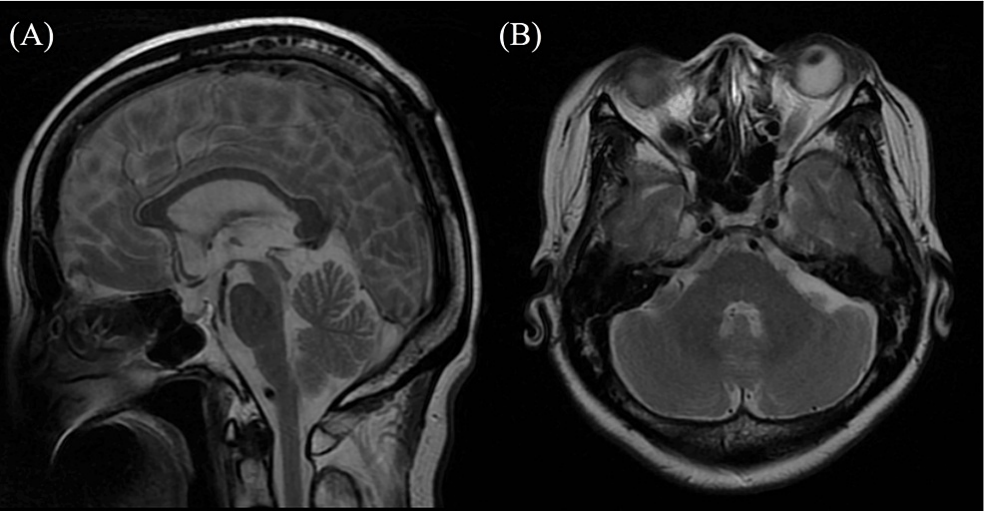
The result of brain magnetic resonance imaging (MRI) (A, B) T2-weighted brain MRI showing that atrophy was unremarkable in the brainstem or cerebellum (A) sagittal section; (B) axial section

A 64-year-old woman, who was previously healthy, developed bradykinesia. She had no family history of PD. At 65 years old, she had difficulty riding on a bicycle due to a problem with lifting her legs. At age 66 years, she developed a tendency to fall backward and visited a previous doctor. Neurological examinations revealed a masked face, positive for Myerson’s sign, mild cogwheel rigidity, and postural tremor (dominant in the left). Rest tremors, brachybasia, and constipation were not noted. Brain magnetic resonance imaging (MRI) revealed no atrophy of the brainstem or cerebellum (Figure 1).

**Figure 2:**
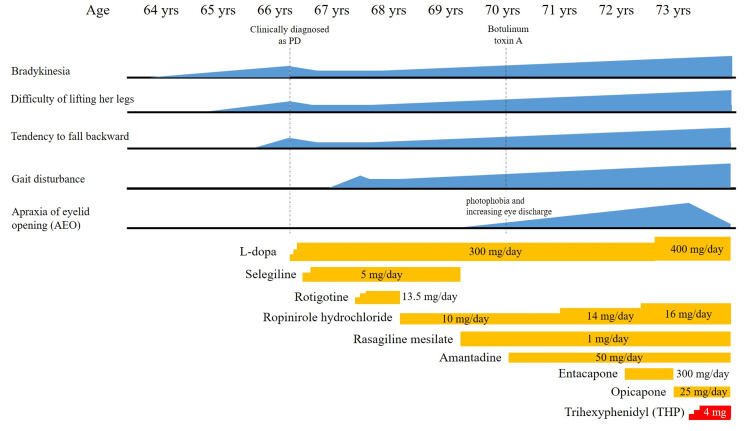
The clinical course of our patient including medication history The clinical course of our patient, including medication history. Her symptoms were improved in the early phase by medications, but the benefit was unremarkable in a subsequent phase. Trihexyphenidyl (THP) is effective for apraxia of eyelid opening drastically.

She did not take nuclear medicine examinations, including 123I-metaiodobenzylguanidine (MIBG) myocardial scintigraphy or dopamine transporter imaging. The L-dopa challenge test improved her symptoms, including bradykinesia; hence, she was clinically diagnosed with PD by a previous doctor, although there was no evidence of nuclear medicine. The clinical course, including medication history, is shown in Figure 2. At age 67 years, gait disturbance developed. Then, her symptoms were exacerbated gradually. Although her medications were dosed up as her symptoms progressed, the improvement in her symptoms was limited. At age 69 years, she took three types of anti-Parkinson medications, including L-dopa 300 mg, selegiline 5.0 mg, and ropinirole hydrochloride 10 mg. Additionally, our patient spent much of the day with her eyelids closed with blepharospasm and she was diagnosed with AEO. In addition, off symptoms appeared. Whereas selegiline was changed to rasagiline 1 mg, her symptoms did not improve, including bradykinesia or AEO. At age 70 years, she was treated with botulinum toxin A (total 24 units) for AEO, but her satisfaction level was low for developing photophobia and increasing eye discharge. At age 72 years, her symptoms were more worsened. Then, she took five types of anti-Parkinson agents, including L-dopa 300 mg, rasagiline 1 mg, ropinirole hydrochloride 14 mg, amantadine 50 mg, and entacapone 300 mg. Due to severe AEO, she could no longer keep her eyes open most of the daytime. For AEO, she used both hands to keep her eyes open. She attempted to adjust her medication as an outpatient, but her motor symptoms, including AEO, did not improve. At age 73 years, she was admitted to our hospital for medication adjustment. On admission, she took five types of anti-Parkinson agents, including L-dopa 400 mg, rasagiline 1 mg, ropinirole hydrochloride 16 mg, amantadine 50 mg, and entacapone 300 mg. Whereas we changed entacapone 300 mg to opicapone 25 mg at first, her symptoms, including bradykinesia or off symptom, were not improved. We added THP from 1 mg up to 4 mg gradually. Depending on the dosage of THP, the duration of opening her eyelids was prolonged. After the dose-up of THP to 4 mg, she spent most of her day with her eyelids open, which provided our patient with an improvement in activity of daily living. She was discharged from our hospital on day 30 after admission.

## Discussion

This report describes a case demonstrating the efficacy of THP for AEO due to Parkinsonism. She developed not only extremity-trunk motor symptoms but also eye symptoms including AEO and blepharospasm. Various antiparkinsonian drugs were ineffective against AEO, except THP. Although our patient was clinically diagnosed as PD by a previous doctor, we seemed that she is more appropriate for a diagnosis of PSP, considering the entire history. The reasons we considered PSP were as follows: (1) tendency to fall backward from early onset, (2) poor improvement for anti-Parkinson agents including L-dopa (but in the early phase, some benefits were noted by medications), (3) no constipation or tremors. Although an accurate diagnosis cannot be made due to a lack of evidence by radiological and nuclear medicine tests, we considered her to be PSP-Parkinsonism, a variant of PSP [4]. AEO and blepharospasm are more common in PSP and MSA rather than PD [5]. In addition, it has been reported that AEO either combined with or without blepharospasm may occur in parkinsonism, especially in PSP [5-9]. Thus, the presence of these ocular symptoms supports PSP rather than PD.

Regarding the etiology of AEO and blepharospasm in PSP, focal dystonia is the most likely cause [10,11]. In most cases of AEO in Parkinsonism, blepharospasm is also seen [5]. Although the pathogenesis of AEO and blepharospasm is less understood, a few hypotheses were suggested based on animal model studies [1]. The nigrostriatal basal ganglia pathway may be involved in the premotor control of eyelid coordination. Thus, it is associated with dysfunction of corticothalamic, basal ganglia, and local cranial neural circuits [12]. Structures involved include sensorimotor cortical regions, substantia nigra pars reticulata, and brainstem motor nuclei (trigeminal blink reflex arc) [1]. In addition, abnormalities in dopaminergic neurotransmission may underlie abnormal eye blinking and eyelid muscle contraction at a biochemical level [1]. It is estimated that involvement of these pathways may lead to poor eyeblink coordination and the development of dystonic movements, leading to AEO and blepharospasm. Therefore, it is more likely to develop AEO and/or blepharospasm in neuromuscular diseases in which these pathways are mainly impaired, including Parkinsonism or Huntington’s disease [12].

THP is a selective muscarinic acetylcholine receptor antagonist, blocking cholinergic activity centrally and peripherally [13]. THP is one of the most used medications among children to improve dystonia [3], owing to its effects on tone reduction and overall functional improvement [14]. The specific anti-dystonia mechanism of THP is less understood, but it is probably associated with its antimuscarinic effect [14]. In knock-in mice with primary dystonia (Dyt1 KI), the muscarinic anti-cholinergic receptor (mAChR) antagonism is specifically required to offset plasticity deficits [14]. Indeed, THP is widely used in some dystonic diseases [14,15]. In addition, as far as we know, two papers have reported that THP is effective for AEO [11,16]. Of them, one report describes AEO as Parkinsonian but idiopathic torsion dystonia [16], but THP may improve AEO via dystonia relief.

Regarding to treatment of AEO by Parkinsonism, it is reported that aripiprazole may be one choice [17]. Authors in this report considered aripiprazole may improve AEO by acting on the dopaminergic or serotoninergic systems [17]. However, aripiprazole acts as an antagonist of dopamine receptors, which might exacerbate Parkinsonian symptoms [17]. Indeed, Fernandez et al. reported that two out of six patients discontinued aripiprazole due to worsening motor symptoms in PD [18]. Based on this concern, we would like to suggest that THP, which was developed as a medication for PD, might be the first choice for AEO by Parkinsonism.

## Conclusions

We presented a case demonstrating the efficacy of THP for AEO due to Parkinsonism. Although other anti-Parkinsonian medications did not improve AEO, TPH improved AEO drastically in our case. The mechanism of the TPH effect on AEO might be an alleviation of dystonia. Whereas aripiprazole has been reported to improve AEO, TPH may be the first choice for AEO in terms of concerns about worsening Parkinson's symptoms by dopamine antagonists. To verify the effect of THP on AEO by Parkinsonism, we need to accumulate more cases.
